# Association of waterpipe smoking and road traffic crashes

**DOI:** 10.1186/1471-2458-10-639

**Published:** 2010-10-23

**Authors:** Soheil Saadat, Mojgan Karbakhsh

**Affiliations:** 1 Research Assistant Professor in Epidemiology, Sina Trauma Research Center, Tehran University of Medical Sciences, Tehran, Iran; 2 Assistant Professor in Community medicine, Sina Trauma Research Center, Tehran University of Medical Sciences, Tehran, Iran

## Abstract

**Background:**

The purpose of this research was to examine whether waterpipe smokers experience increased risk of motor vehicle crashes.

**Methods:**

In a telephone survey, a random sample of Iranian drivers were asked to report their age, gender, vehicle age, whether their vehicles were equipped with anti-lock braking system (ABS), average daily drive time (DDT), whether they smoked cigarette or waterpipe, whether they had diabetes mellitus (DM), number of traffic crashes during the last calendar year and whether the crash involved a pedestrian or another vehicle.

**Results:**

A total of 2070 motor vehicle owners with the mean age of 41.6 ± 11.45 were interviewed. The annual incidence of Road Traffic Crashes (RTC) was 14.9%; 14.0% involved a collision/s with other vehicles and 0.9% with pedestrians. There was an association between the RTC and male gender, DDT, being a cigarette smoker, being a waterpipe smoker and DM in univariable analysis. The association between RTC and being a waterpipe smoker and also cigarette smoker was significant in multivariable analysis after adjustment for DDT.

**Conclusions:**

Being waterpipe and/or cigarette smoker and DDT were the independent predictors of the number of traffic crashes in Poisson regression model. If the increased risk of RTC among waterpipe or cigarette smokers is seen in other studies, it would be beneficial to promote tobacco cessation and control strategies through injury prevention initiatives.

## Background

**Figure 1:**
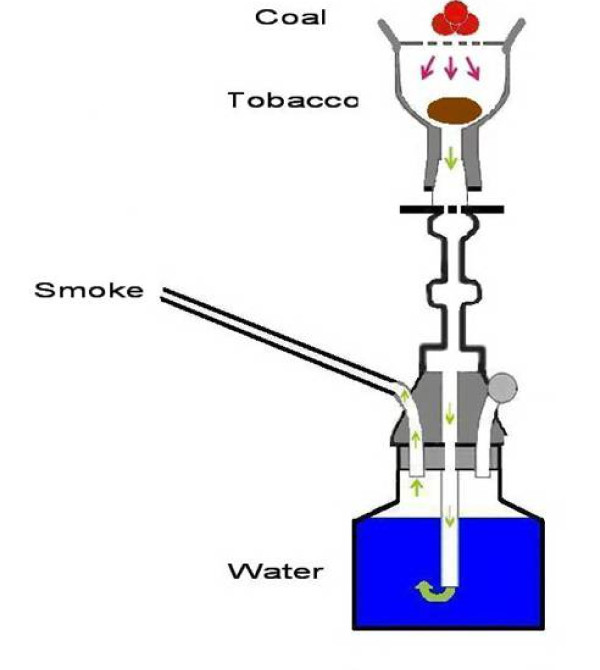
**A schematic presentation of waterpipe and its essential components**.

Road traffic Injuries (RTI) are a major global public health and development problem that are projected to worsen in the following years. Over 50% of deaths due to RTI are among young adults in the age range of 15-44 years [1]. Although RTI is a major health problem per se, its combination with smoking practice appears to be more alarming from preventive standpoint. Smokers are not only at risk for the chronic diseases such as cancer and respiratory diseases, but also they experience increased risk of fatal and non-fatal road traffic crashes (RTC) compared to non-smokers [2,3]. Waterpipe smoking is a re-popularized method of tobacco use throughout the world with an estimated 100 million daily smokers [4]. It is also called hooka, hubble-bubble, boory, goza, shisha- meaning "glass" in Arabic which refers to the glass base-, narghile -from the Persian word meaning "coconut" as the early waterpipes were made of coconut shells-, and nowadays qalian (in Iran) [5,6] (Fig1). Originating in India in the early 1700s, waterpipe has been a traditional way of smoking in the Eastern Mediterranean Region for decades [7,8]. After a decline in popularity in 1980 [5], waterpipe is now widespread more than ever especially among young people [5,9-13] and even among adolescents [14,15] and pregnant women [16,17]. Recent evidence shows that waterpipe is not just a regional health problem anymore with the lifetime use similar to lifetime cigarette use among a random sample of American University students [18]. Now the fruit- and sweet-flavored hookahs have found their ways to restaurants and nightclubs in major US cities [19] justifying the rates as high as 50% lifetime prevalence rate and 20% past 30-day use rate among first-year college students in some states [20]. Other reports from the United States[21,22], United Kingdom[23], Estonia[24], Australia[25], Ukraine[26], Germany[27], Brazil[28], Canada[29], and Korea[30] also substantiate the evidence for this global public health threat. Generally speaking, the burden seems to be huge and increasing due to smoking-related harms, unrealistic risk perception and socio-cultural acceptability [5,31,32].

In this time of injury epidemic[33] and growing waterpipe use, no previous investigations have answered the question of whether waterpipe is a contributor to this rising injury burden. The purpose of this research was to examine whether waterpipe smokers experience increased risk of motor vehicle crash.

## Methods

In this cross-sectional study, a telephone survey was arranged over a random sample of Iranian drivers. The telephone numbers of drivers were collected from Iranian Central Insurance Organization which has the information of all drivers who have purchased the compulsory third party vehicle insurance contracts. In Iran, third party vehicle insurance coverage is mandatory for all vehicles. Thus, as soon as a person buys an automobile and insures it, the insurance company reports the customer's record to the Central Insurance Organization.

A total of 3000 car owners were selected randomly from all over the country. They were informed about the general objectives of the research and were asked if they were willing to participate. Of the 3000 contacted car owners, 346 were excluded as they declared they just owned the car and did not drive. Of the remaining 2654 drivers, 2070 were willing to participate (response rate: 78.3%).

These drivers were asked to report their age, gender, vehicle age, whether their vehicles were equipped with anti-lock braking system (ABS), average daily drive time (DDT), whether they smoked cigarette or waterpipe (we did not mean smoking during driving, but habitual smoking, regardless of situation and time), whether they had diabetes mellitus (DM), number of traffic crashes during the last calendar year and whether the crash involved a pedestrian or another vehicle. To facilitate recalling the number of crashes during the study period, the survey was performed at the last ten days of Persian calendar year.

Interviewers were trained for this telephone interview and a detailed guideline was provided indicating how to explain about the research nature of the study and ask every specific question. The interview team consisted of four interviewers and a supervisor who was responsible for quality assurance of interviews according to the provided guideline.

If a target driver was not accessible at the first attempt, the interviewers tried again for two more other times and then in the case of unavailability, substituted him/her with the next person in the list of randomly selected drivers.

Data was entered twice and any discrepancy was sought referring to original paper records. Crude odds ratio (OR) was calculated to compare collisions between smokers and non-smokers; Mantel-Haenszel method was used to calculate adjusted odds ratio in tabular analysis. A Poisson regression model was used to assess the association of number of collisions with being a smoker while controlling the effect of age, gender, vehicle age, ABS, DDT and DM. STATA 8 software was used for data analysis.

## Ethical Approval

The proposal of this research was approved by the ethical committee of Sina Trauma Research Center affiliated to Tehran university of Medical Sciences.

## Results

A total of 2070 motor vehicle owners were interviewed over the phone. About 14.9 (95% CI: 13.4 - 16.5) percent (n = 308) reported at least one RTC during the last calendar period: 14.0% involved collision/s with other vehicles and 0.9% involved collisions with pedestrians (Table 1).

**Table 1:** Frequency distribution of study participants according to the number of collisions during the preceding year

N of collisions with another vehicle N (%)	N of collisions with Pedestrian N (%)		Total
	1	0	
3	0 (0)	2 (0.1)	2 (0.1)
2	0 (0)	44 (2.1)	44 (2.1)
1	2 (0.1)	244 (11.8)	246 (11.9)
0	16 (0.8)	1762 (85.1)	1778 (85.9)
Total	18 (0.9)	2052 (99.1)	2070 (100)

The basic characteristics of responders who reported at least one traffic crash in comparison with those who did not report any is displayed in table 2.

**Table 2:** The basic characteristics of responders who reported to have at least one traffic crash during the preceding year in comparison with those who did not report any crashes

**Variable**	**Levels**	**Any RTC 308 (14.9%)**	**No RTC 1762 (85.1%)**	**Total N (%)**	**Odds Ratio(0.95 CI)**	**P value**
**Gender**	Male	266 (15.4)	1459 (84.6)	1724 (83.3)	M/F: 1.61 (1.10 - 2.43)	0.012
	Female	34 (10.1)	301 (89.9)	346 (16.2)		
	Missing	8	2			
**daily drive time (DDT)**	>12	0 (0.0)	52 (100.0)	52 (2.6)	Mantel-Hanszel OR: 1.03 (0.91-1.17)	0.011
	8.1-12	15 (11.3)	118 (88.7)	133 (8.4)		
	3.1-8	103 (21.5)	376 (78.5)	479 (30.3)		
	1.1-3	127 (14.4)	755 (85.6)	882 (43.8)		
	≤1	60 (12.7)	410 (87.3)	470 (23.3)		
	Missing	3	51			
**vehicle age**	≥ 5 Yrs	77 (16.9)	378 (83.1)	455 (22.4)	1.22 ( 0.91 - 1.63)	0.168
	<5 Yrs	226 (14.3)	1353 (85.7)	1579 (77.6)		
	Missing	5	31			
**ABS Brakes**	No	191 (10.5)	1636 (89.5)	1827 (92.7)	0.64 (0.39 - 1.09)	0.070
	Yes	22 (15.4)	121 (84.6)	143 (7.3)		
	Missing	95	5			
**Cigarette smoking**	Yes	82 (20.9)	311 (79.1)	393 (19.3)	1.68 (1.25 - 2.25)	<0.001
	No	223 (13.5)	1424 (86.5)	1647 (80.7)		
	Missing	3	27			
**Water pipe smoking**	Yes	42 (27.8)	109 (72.2)	151 (7.4)	2.41 (1.61 - 3.57)	<0.001
	No	260 (13.8)	1629 (86.2)	1889 (92.6)		
	Missing	6	24			
**Diabetes**	Diabetic	14 (25.9)	40 (74.1)	54 (2.6)	2.07 (1.02 - 3.97)	0.030
	Non diabetic	220 (14.4)	1304 (85.6)	1524 (73.6)		
	Don't know	74 (15.0)	418 (85.0)	492 (23.8)		
**Driver's age**		41.8 (± 11.53)	40.7 (± 11.05)	41.6 ± 11.45		0.881

The association between gender and the involvement in a crash was not statistically significant after adjustment for DDT (Mantel-Haenszel OR: 0.63 - 2.01). Diabetic drivers were older (50.0 ± 10.22) than non diabetics (41.2 ± 11.37) and the difference was statistically significant (P < 0.001). The association between DM and RTC was not statistically significant after adjustment for DDT (Mantel-Haenszel OR: 0.92 - 3.26). Being cigarette smoker showed a significant association with having a crash even after adjustment for DDT (Mantel-Haenszel OR: 1.02 - 2.11).

The association between being a waterpipe smoker and having a crash also remained statistically significant after adjustment for DDT (Mantel-Haenszel OR: 1.27 - 3.35).

The result of Poisson regression representing the association of number of traffic accidents with being cigarette smoker, being waterpipe smoker, DDT, gender and DM is displayed in table 3.

**Table 3:** Factors associated with the number of crashes in Poisson model

**Variable**	**Coefficient**	**Standard Error**	**P Value**
**Being a cigarette smoker**	0.34	0.159	0.035
**Being a waterpipe smoker**	0.44	0.216	0.042
**Driving duration in a day**	0.18	0.066	0.007
**Being diabetic**	0.33	0.298	0.274
**Gender**	-0.05	0.244	0.825
**Model constant**	-2.13	0.478	0.000

The variables remaining in the Poisson regression model reduced to those represented in table 4 after removing DM and gender which failed to achieve statistical significance in the multivariable analysis.

**Table 4:** Poisson regression coefficients after removing DM and gender

**Variable**	**Coefficient**	**Standard Error**	**P Value**
**Being a cigarette smoker**	0.33	0.152	0.029
**Being a waterpipe smoker**	0.49	0.201	0.014
**Driving duration in a day**	0.15	0.064	0.022
**Model constant**	-2.14	0.168	0.000

## Discussion

Our study is among the first to show the higher risk of RTC in waterpipe smokers, yet the mechanisms of this finding need to be studied further. The mechanism(s) might be partially similar to cigarette smoking. The association of smoking and RTC has been demonstrated in some prior studies. Studies from Spain and also the United States have shown smokers to have a 50 percent higher risk of RTC than nonsmokers[34,35]. In the study from the US which was on subjects attending a driving safety course, this surplus remained even after controlling for the effect of age, education, alcohol consumption and driving experience[35]. Another study from Canada showed that 30-39 year old males who had been at-fault in crashes were 1.5 times more likely to be smokers[36]. Other studies have shown this risk to be higher for smoking while driving. In a Montreal study, drivers involved in MVCs resulting in injury or death were 1.75 more likely to have been smoking at the time of crash than matched controls[37]. However, our study did not focus on smoking while driving, but being a smoker at all.

The relationship between smoking and RTC seems to be far from distraction and carbon monoxide effect on the driver as the risk exists regardless of whether drivers refrain from smoking while driving or not[34]. Other mechanisms which have been implicated are as follow: cognitive impairments secondary to chronic nicotine exposure, risk taking and sensation seeking behaviors [38,39], smoke-induced eye blurring and cough and the resultant fatigue[40,41] and even decreased vision of smokers due to deposited smoke on the automobile windshield[36]. Prior injury history and risky behaviors such as seat belt non-use have also been reported to be more common in smokers[2].

The prevalence of waterpipe and cigarette smoking might be different in diabetics compared with non-diabetics, as diabetics are usually prohibited from smoking to decrease the risk of heart ischemia. Therefore, we adjusted the association of RTC and smoking for the effect of DM and we noted that the association was in place regardless of DM.

It has been shown that DM drivers have higher rates of RTC[42,43]. We observed the same result in univariable analysis but the association failed to achieve statistical significance after adjustment for DDT. Although this study was not designed to study the association of DM and the risk of RTC, it seems that DDT modifies such an association. A reason for increased risk of RTC in DM drivers is hypoglycemia [43].The risk of hypoglycemia may be increased by longer driving. If this is the case, limiting DDT for DM drivers should be considered in order to control the risk of RTC. This needs to be addressed in future studies.

The percentage of RTCs among car owners reported by drivers (13.4% - 16.5%) is similar to national reports of the Iranian Central Insurance Organization. During the Persian year of 1386 (20 March 2007 - 19 March 2008), 11,534,657 third party insurance contracts were sold in Iran and in 1,538,593 cases (13.3%) it resulted in claims for compensation[44]. Assuming that drivers who had a mild TRC did not refer to Insurance companies to claim for compensation (to keep their records clean and avoid time consuming bureaucratic processes), the slightly higher rate of RTCs reported by car owners in our study could be considered consistent with above mentioned national statistics. The prevalence of smoking was similar to other studies [45]. These similarities make it unlikely that the study sample is different from target population.

We noted lower incidence of RTCs reported by female drivers. However, the association between gender and having a crash did not remain statistically significant after adjustment for DDT. Longer DDT may play a central role in increased total RTCs among male drivers. Most of professional drivers (taxi or truck drivers) in Iran are male and they are expected to drive for longer durations of time than other drivers. In addition, we see that in Iran, men generally tend to drive more than women even if their jobs are not related to driving. In our research, adjustment for the DDT removed the potential association with gender. Throughout the world, numerous studies have already demonstrated that men have higher road traffic related death and mortality rates. Nevertheless, the association between gender and involvement in road crashes is less consistent in different studies. This sounds tangible as being injured in a road crash is a function of risk, risk exposure and also the severity of exposure. For instance, in a study based on crash involvement rates per vehicle-mile of travel in the US, male drivers had higher risks of experiencing fatal crashes, while women had higher rates of involvement in injury crashes and all police-reported crashes [46]. Another study also demonstrated young males to be more likely than young females to contribute to crash deaths [47]. This is while another study from Slovenia has highlighted the increasing role of women in RTCs [48].

During the tabular analysis, adjustment for the effect of DDT diminished the association between RTC and most of the independent variables of this study. However, the association remained statistically significant in the case of cigarette smokers and waterpipe smokers. We conclude that the association between these two variables and the RTC is more stable compared to DM, gender, ABS, driver's age and car age.

The prevalence of RTCs in drivers who reported smoking both cigarette and waterpipe was more than those who reported only waterpipe smoking which was itself more than those who smoked only cigarette. The association of RTC with waterpipe smoking was independent from cigarette smoking and also stronger than that as it is evident in tables 2,3 and 4.

Some facts increase the policy implications of this study. Iran has one of the highest death rates due to road traffic crashes throughout the world (44 per 100,000) with the highest Disability Adjusted Life Years (DALYs) among young people(age group of 25 - 34 years), with male to female ratio of about four to one[49]. A total of 209,923 deaths from RTCs have been recorded in Iran from 1997 to 2006[50].

Our study had some limitations. In a telephone survey, people who do not have a telephone line have no chance to appear in the sample. This potential shortcoming does not seem to be a major source of bias in this study as in Iran, nearly all people who own a car, have also telephone at home/work. As another limitation, we were not able to verify the information provided by participants from any other sources.

## Conclusion

This study showed the independent association between out-of-the wheel waterpipe smoking and having road traffic crashes and also the number of crashes. Further research is necessary to show the exact mechanisms that render waterpipe smokers to have higher number of crashes. In addition, the association shown in this manuscript is not necessarily a causative one. For instance, "clustering of high risk behaviors" in people can be proposed as a potential explanation: people who smoke waterpipes are more likely to be more careless drivers; as they might prefer driving with high speeds and driving under the influence of drugs and alcohol. Unfortunately, the data collected in this study are not sufficient to test this hypothesis of risk factor clustering in drivers who smoke waterpipes.

If the increased risk of RTC among waterpipe or cigarette smokers is seen in other studies, it would be beneficial to promote tobacco cessation and control strategies through injury prevention initiatives.

## Competing interests

The authors declare that they have no competing interests.

## Authors' contributions

SS proposed the idea for this study, designed the project, analyzed the data and participated in drafting and finalizing the manuscript. MK participated in data analysis and manuscript preparation and submission. Please indicate that both authors read and approved the final manuscript.

## Pre-publication history

The pre-publication history for this paper can be accessed here:

http://www.biomedcentral.com/1471-2458/10/639/prepub
